# Intracellular traffic and polarity in brain development

**DOI:** 10.3389/fnins.2023.1172016

**Published:** 2023-10-04

**Authors:** Martina Polenghi, Elena Taverna

**Affiliations:** Human Technopole, Milan, Italy

**Keywords:** trafficking organelles, brain development, Golgi apparatus, neurodevelopmental disorders, epithelial polarity, neural stem and progenitor cells

## Abstract

Neurons forming the human brain are generated during embryonic development by neural stem and progenitor cells via a process called neurogenesis. A crucial feature contributing to neural stem cell morphological and functional heterogeneity is cell polarity, defined as asymmetric distribution of cellular components. Cell polarity is built and maintained thanks to the interplay between polarity proteins and polarity-generating organelles, such as the endoplasmic reticulum (ER) and the Golgi apparatus (GA). ER and GA affect the distribution of membrane components and work as a hub where glycans are added to nascent proteins and lipids. In the last decades our knowledge on the role of polarity in neural stem and progenitor cells have increased tremendously. However, the role of traffic and associated glycosylation in neural stem and progenitor cells is still relatively underexplored. In this review, we discuss the link between cell polarity, architecture, identity and intracellular traffic, and highlight how studies on neurons have shaped our knowledge and conceptual framework on traffic and polarity. We will then conclude by discussing how a group of rare diseases, called congenital disorders of glycosylation (CDG) offers the unique opportunity to study the contribution of traffic and glycosylation in the context of neurodevelopment.

## Introduction

Mammalian brain, and the human brain in particular, is a complex organ that needs high-level network-like organization for proper functioning. From an evolutionary point of view, the neocortex is the youngest part of the cerebral cortex responsible for higher order cognitive functions. The neocortex develops through a very precise sequence of symmetric and asymmetric division of neural progenitor cells (NPCs) (Noctor et al., 2004). NPCs proliferate or self-renew, and they generate—in a direct or indirect way—neurons. Neurons then migrate radially to the basal part of the cortex where they form, along with glial cells, the six-layered neocortex (Taverna et al., 2014). Neocortex development and evolution are linked to an increase in NPCs number and diversity (Rakic, 1995; Fish et al., 2008). Of note, NPCs morphological and functional diversity is mainly driven by difference in cellular polarity, defined as differential localization in space and time of cellular components and compartments (Arai and Taverna, 2017).

In this review we will first provide an overview on (i) NPCs classification and diversity (ii) the role of polarity in NPCs and discuss (iii) traffic from the endoplasmic reticulum (ER) and the Golgi apparatus (GA) in NPCs and (iv) how traffic diseases can inform us on the functional role of traffic in NPCs.

### Neural stem cell types in the developing brain

**Figure 1:**
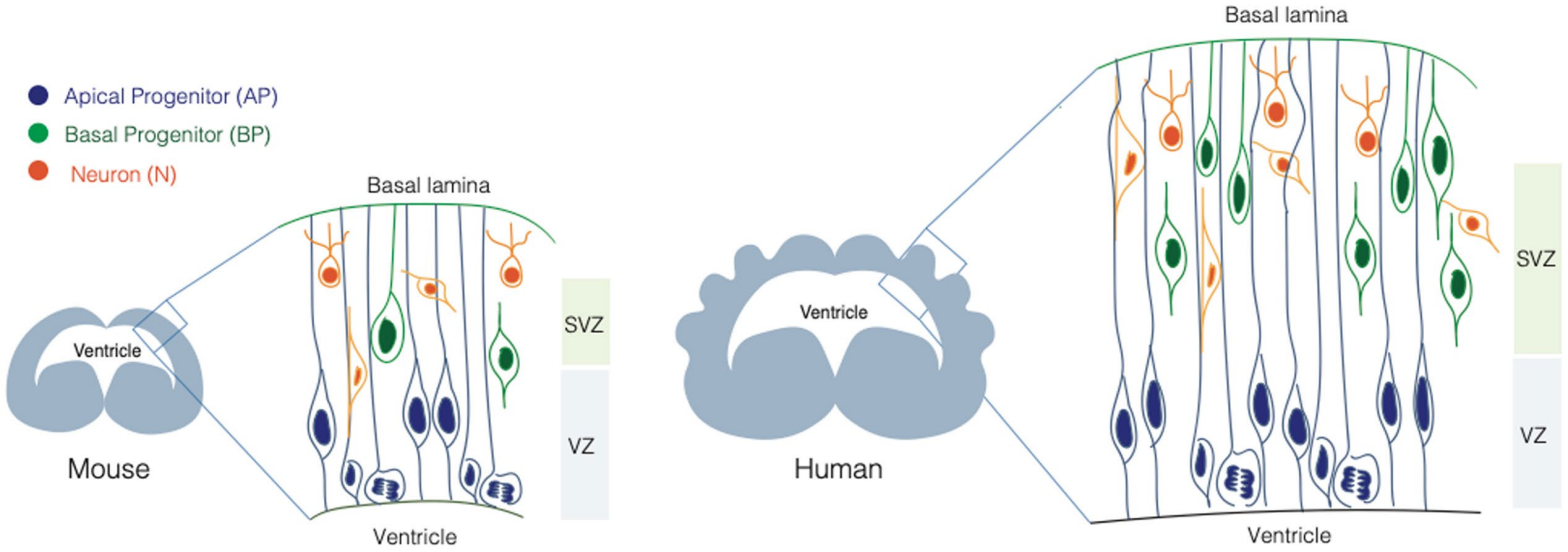
Neural stem cells in the developing mouse and human brain. Scheme illustrating the neural stem cell identity and composition of the mouse (left) and human (right) developing brain. Apical progenitors (APs, in blue), are epithelial cells that occupy the ventricular zone (VZ) and they span the whole thickness of the developing neocortex. Basal progenitors (BPs, in orange) occupy the sub-ventricular zone (SVZ) and they show different morphologies and polarity features. BPs are the main source of neurons (N, in green) in the developing mammalian brain.

Neurons populating the neocortex are generated during embryonic development from two main classes of NPCs: apical progenitors (APs) and basal progenitors (BPs) (Figure 1). The broad classification between APs and BPs reflects the location where they undergo mitosis (Taverna and Huttner, 2010; Taverna et al., 2014). APs reside in the apical-most region of the developing neocortex, called ventricular zone (VZ) and they undergo mitosis at the apical surface of the VZ. BPs reside in a more basal area called subventricular zone (SVZ), where they undergo mitosis. APs and BPs show strikingly different cell biology and cellular architecture and make differential use of polarity cues, which is intimately linked to their function and balance between their proliferation and differentiation potential (Fietz and Huttner, 2011).

#### Apical progenitors

APs are epithelial cells, with a very small apical plasma membrane (1%–2% of the total plasma membrane) lining the ventricle, and a very elongated basolateral plasma membrane reaching the basal lamina (Figure 1). APs elongation can be extreme, as in the case of the developing primate brain, where the distance between the apical surface and the basal lamina can be up to several millimeters. APs are further subdivided into neuroepithelial cells (NE) and apical radial glia cells (aRGs). NE are the founder cells of CNS, they are present during early neurogenesis, they occupy the VZ and they proliferate by dividing symmetrically, expanding the pool of NPCs (Götz and Huttner, 2005). aRGs appear at mid-neurogenesis, they occupy the VZ and contact the basal lamina via their basal process, that spans the SVZ and the forming cortical plate (Taverna et al., 2014). APs undergo interkinetic nuclear migration (INM). During INM the nucleus moves from the apical to the basal part of the VZ (and back) in concert with cell cycle progression, so that mitosis always happens at the apical surface, and S-phase in the basal most part of the VZ (Götz and Huttner, 2005; Taverna and Huttner, 2010; Taverna et al., 2014). INM confers to the VZ a pseudostratified appearance.

The division of APs is typically asymmetric self-renewing and generate a BP, that delaminates from the ventricular surface by losing the apical attachment. Delamination is strongly reminiscent of epithelial-to-mesenchymal transition, that is known to be mediated by a reorganization of the intracellular architecture (Wilsch-Bräuninger et al., 2012; Kawaguchi, 2021).

#### Basal progenitors

BPs are a very heterogeneous class of progenitors, that divide basally in the SVZ (Figure 1). BPs differ in proliferative capacity, molecular landscape, and they typically lack apical attachment (Kriegstein and Alvarez-Buylla, 2009; Borrell and Reillo, 2012; Kalebic and Huttner, 2020). The occurrence and type of their basal polarity cues allow a further classification between intermediate progenitors (IPs) and basal radial glia cells (bRGs) (Florio and Huttner, 2014; Namba and Huttner, 2017; Kalebic and Huttner, 2020). IPs lack both apical and basal attachment and polarity, while bRGs can retain basal attachment, and can feature basal and/or apical polarity cues. The combinatorial presence of polarity cues is likely to expose BPs to a different subset of environmental signals compared to the ones reaching APs. The link between these cues and cell proliferation/differentiation potential is a very active area of research in the field of cell biology of neurogenesis (Kalebic and Huttner, 2020).

### Polarity in neural stem cells

The term polarity refers to the asymmetric distribution of cellular features, subcellular compartments and molecular components within the cell (Macara and Mili, 2008). Molecular and subcellular polarity allows compartmentalization of functions and it is instrumental for the ability of the cell to interact with and respond to the extracellular environment (Arai and Taverna, 2017). Notably epithelial cells are polar cells with at least two distinct compartments: an apical compartment facing a lumen and a basal compartment in contact with the basal lamina. Compartmentalization of function is a shared feature between epithelial cells and neurons, that developed dedicated compartments to receive as well as to send signals from and to the extracellular environment or other cells they are in contact with. Changes in polarity features of single cells are often accompanied by whole tissue remodeling through migration of cells to different destinations, a process that can be observed from very early stages of development when cell identity and fate are determined by cell localization and by the repertoire of signals the cell is exposed to (Pinheiro and Heisenberg, 2020). But what about neural stem cells? In the context of brain development the link between cell identity and polarity is particularly intriguing as AP-to-BP fate switch, the central fate change in brain ontogeny and phylogeny, entails a major change in cell polarity.

#### Polarity to sense and integrate signals from the extracellular environment

Apical progenitors possess an apical process contacting the apical surface and a basal process touching the pial surface. Differently, most BPs delaminate from the apical surface and only maintain contact with the basal membrane though some cells lose the basal process too. Delamination exposes BPs to only a subset of signals coming from the external environment, compared to the environmental cues that reaches both extremities of APs. The lumen of the ventricle is filled with CSF (Lehtinen et al., 2011) with signaling molecules such as FGF, IGF, Shh, retinoic acid, BMP and Wnts (Long et al., 2016; Wang et al., 2016): the action of these molecules is thought to happen via interaction with their receptors which are indeed localized at the plasma membrane of the apical process. An example of such interaction is provided by megalin, a glycoprotein enriched at the apical surface in the neuroepithelium, that interacts with BMPs and Shh; megalin mediates the endocytosis of Bmp4 and subsequent degradation, allowing Shh expression. Megalin KO mice display abnormal forebrain development following the increase in Bmp4 and loss of Shh. These mice show loss of ventrally derived oligodendroglial cells as well as interneuronal population. Megalin-triggered signaling pathway directly connects the extracellular environment with changes at the gene expression level in these neuroepithelial cells ultimately affecting brain developmental pathways (Willnow et al., 1996; Spoelgen et al., 2005).

At the level of the basal lamina APs receive signals from the meninges and the blood vessels that start developing. Interestingly, signals coming from meningeal cells, such as retinoic acid, were shown to have a role in cell identity switch from AP to BP (Siegenthaler et al., 2009; Janesick et al., 2015).

In the basal compartment, crucial players are laminin α2 and α4 and integrins (Taverna and Huttner, 2003; Fietz et al., 2010; Long et al., 2016; Kalebic et al., 2019). Integrins play a structural role by mediating the interaction of the basal process with the basal lamina as well as a functional role as they were shown to maintain the AP pool during development. Integrin αvβ3 is expressed by BPs and regulates their proliferation via its thyroid hormone receptor function. Loss of integrin β1 is accompanied by reduction in neural progenitor proliferation and their responsiveness to EGF, FGF and NGF signaling (Leone et al., 2005; Long and Huttner, 2019). BPs, lacking an apical contact, will receive a different subset of signals from the basal environment or from the surroundings cells in the developing neocortex. Interestingly, integrin β1 is involved in bRG proliferation and expansion (Kalebic et al., 2019), a function tightly linked to the morphological features of bRG itself. These data suggest interesting avenues of investigations on how single neural stem cells do integrate signals during developmental time and in the tissue space, further expanding the concept of stem cell niche to the subcellular scale.

In conclusion, recent data suggest that cell polarity ultimately results in a differential way of sensing, integrating and responding to signals from the extracellular environment, which will influence cell identity. Of note, polarity also offers the structural and architectural basis for asymmetric partitioning of cellular components during cell division.

#### Polarity and cell division

NPCs undergo either a symmetric or an asymmetric division: in the case of symmetric divisions NPCs divide and produce two daughter cells with the same identity of the mother cell (AP → AP + AP, proliferative) or they can differentiate in two neurons (AP → N + N, consumptive). In the case of asymmetric divisions instead, one daughter cell maintains the same identity of the mother cell while the other will acquire a different fate (AP → AP + BP, self-renewing). The balance between these divisions is tightly controlled and alterations of this process lead to neurodevelopmental diseases, as in the case of microcephaly (Carpentieri et al., 2022). The degree of fate asymmetry can be driven by the asymmetric partitioning of cell biological components in the dividing mother cell.

For example, in *Drosophila*, Par-complex proteins, restricted to the apical domain, can either influence the daughter cells fate through indirect interaction with the spindle pole, or by restricting the transportation of proteins such as Miranda, Numb and Prospero selectively to the basal pole of the cell. Besides the well-known antagonism between Numb and Notch signaling and their involvement in maintaining cell identity vs. cell fate switch, Zhou et al. (2007) investigated the interaction between Numb and ACBD3 where the concerted presence of both proteins in the cytoplasm following Golgi apparatus disassembly during mitosis, leads to self-renewal pathway to be chosen by the dividing apical progenitor, at the expenses of neurogenesis.

Furthermore, different studies have demonstrated that inheritance of the apical membrane or of the basal membrane, has a role in driving the proliferation vs. differentiation decision. When having a perfectly apicobasal cleavage plane, the two daughter cells can inherit a portion each of the apical membrane, producing two progenitors that keep proliferating (symmetric proliferative division); conversely, a cleavage plane bypassing the apical PM will result into an asymmetric partitioning of cell biological components and in turn to an asymmetry in cell fate (Kosodo and Huttner, 2009; Taverna et al., 2016; Ayala and Colanzi, 2017). Given the central role of membrane receptors in sensing signals from the outside environment, one might speculate that differential inheritance of membrane(s) (apical and/or basal) might change, at least immediately after division in the G1 phase, the types of signals received by the cell, or the degree of signal integration. Intriguingly, G1 phase was reported as the cell cycle phase when developmental genes responsible for fate specification and switch are activated (Dalton, 2013, 2015).

Taken together, the available data call for a better understanding of the plasma membrane composition, biogenesis and dynamics in neural stem cells. Decades of seminal work on neurons has shown that membrane traffic is one of the main cell biological processes shaping the composition and hence function of highly polarized cells (Bentley and Banker, 2016; Britt et al., 2016). We will here summarize and discuss the current status of knowledge on the role of membrane composition and traffic in neural stem cells, with a focus on the ER and GA.

### Polarity and intracellular architecture

#### Plasma membrane

**Figure 2:**
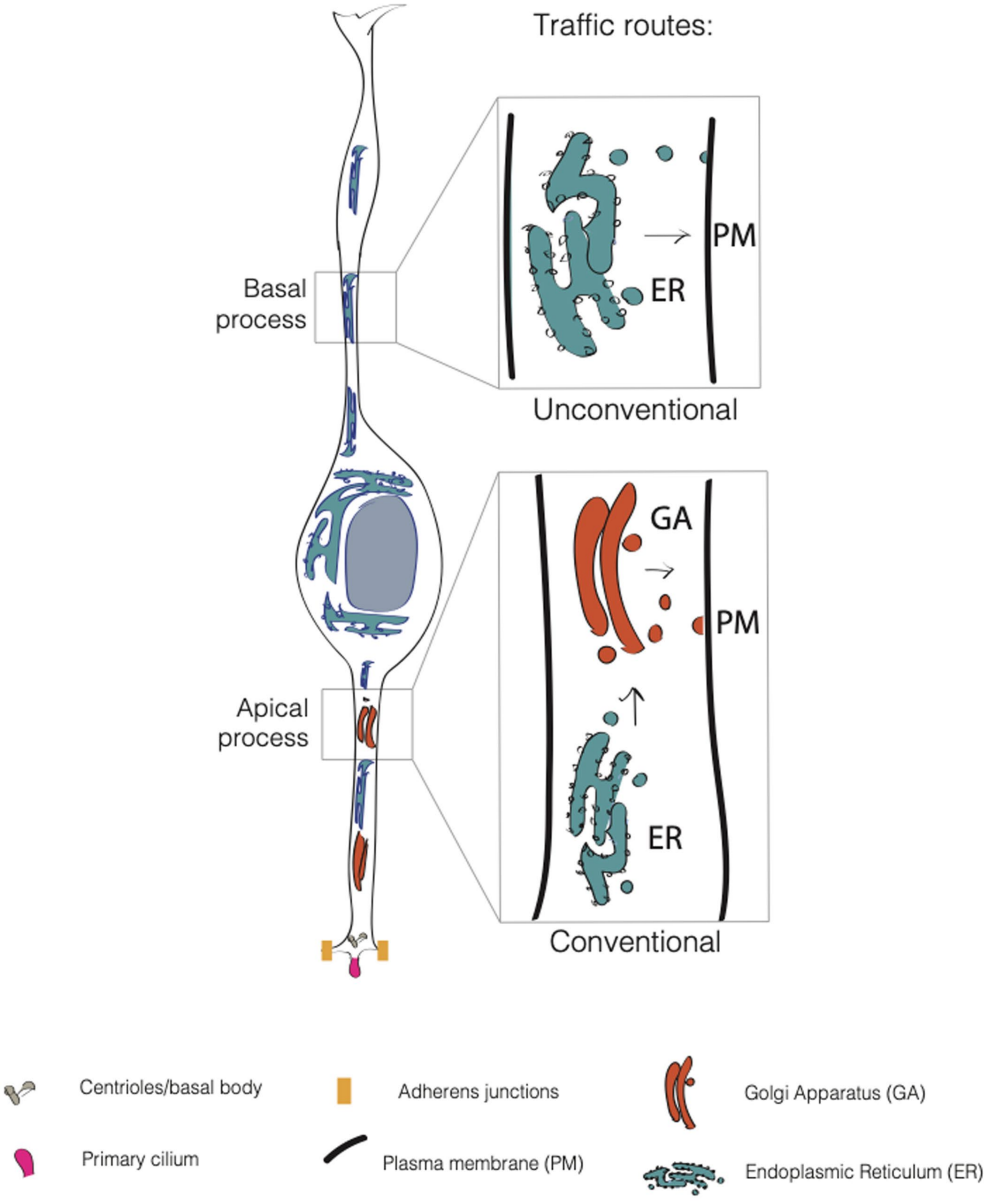
Conventional and unconventional trafficking routes in APs. In apical progenitors (APs), the apical process contains both the endoplasmic reticulum (ER, in blue) and the Golgi apparatus (GA, in dark orange). In contrast, the APs’ basal process contains only the endoplasmic reticulum. Consequently, GA-derived glycans are asymmetrically distributed in APs, as they are enriched only in the apical process. This suggests that the apical process relies on conventional traffic routes, while the basal process relies mainly on unconventional traffic routes.

As all epithelial cells, APs feature an apical and a basolateral plasma membrane (PM) (Figure 2). The apical plasma membrane is subdivided into a planar portion and in the ciliary membrane, a specialized part of the apical plasma membrane that surrounds and delimits the cilium. Paridaen et al. (Paridaen et al., 2013; Paridaen and Huttner, 2014) elegantly showed that the ciliary membrane is internalized, partitioned during mitosis and inherited by one of the two daughter cells. The cell inheriting the ciliary remnant is more likely to have the fate of the mother cell and may in this way respond earlier to extracellular signaling from CSF (Wilsch-Bräuninger et al., 2012). These data suggest the idea that portions of the plasma membrane might endow the cell with the ability to differentially respond to extracellular signals, and again poses the interesting question as to which components and biochemical features confer unique properties to this small portion of the apical plasma membrane. The basaloteral plasma membrane of APs is incredibly extended and traverse several functionally distinct zones, such as the SVZ and the CP. As for BPs, work conducted in bRGs (Kriegstein and Alvarez-Buylla, 2009; Fietz and Huttner, 2011; Borrell and Götz, 2014; Kalebic and Huttner, 2020; Kalebic and Namba, 2021) shows an astonishing level of structural and functional polarization and specialization. This is particularly clear for the apical and basal-directed processes of bRGs, whose presence correlates with the proliferative potential of the cell. The question arises as to which cell biological mechanisms are responsible for the structural, biochemical and functional specialization of the plasma membrane. In this review, we will focus on intracellular traffic.

#### Conventional and unconventional trafficking routes

Lipids and proteins destined to the PM are first processed in the endoplasmic reticulum (ER) where a mannose-rich chain of sugars is added on asparagine residues, in a process called N-glycosylation. The maturation and elongation of the sugar chain is then operated in the GA where discrete units of monosaccharides are added to the protein (or lipid). An alternative to N-glycosylation is O-glycosylation, where sugars are added to the OH group of a serine or a threonine (D’Souza et al., 2021). The trafficking route ER → GA → PM is referred as to conventional secretion. This route is sometimes replaced by an alternative route that bypasses the GA (ER → PM), and that is referred to as the unconventional secretory pathway (USP) (Rabouille et al., 2012; Hanus et al., 2016; Nickel and Rabouille, 2018) (Figure 2). The unconventional routes have been extensively explored in flies, and, of note for this review, in neurons. Bowen et al. (2017) found that AMPA-type glutamate receptor GluA1 and neuroligin undergo ER processing and then accumulate in recycling endosomes at the level of dendrites and spines before being translocated to the plasma membrane. This was found to occur even upon disruption of GA, that implies the presence of an alternative pathway for cargo delivery of proteins that bypasses the GA. In another work, Hanus et al. (2016) showed that numerous synaptic adhesion proteins, surface neurotransmitter receptors, voltage-dependent ion channels and growth factor receptors are largely N-mannose rich, lacking a complex sugar signature feature of Golgi processing. Nevertheless, these proteins are fully functional suggesting that USP is more common than previously thought.

The two classes of glycans can be recognized by concanavalin A (ConA), a lectin that binds to high mannose type glycans derived from the ER, and WGA that binds the complex type glycans derived from Golgi processing (Taverna et al., 2016). The intrinsic property of the glycosylation as a sequential process, already adds a complexity and heterogeneity to all possible modifications that can be added to proteins and lipids, without considering the other post translational modifications, such as sialylation and fucosylation which can be operated by Golgi and other PTMs that can be added before and after the Golgi processing. The fact that cells can also exploit different processing pathway elevates even more the complexity of the glycans and of plasma membrane composition.

### The endoplasmic reticulum

The ER is functionally and morphologically divided in two parts: the rough endoplasmic reticulum (RER) and the smooth endoplasmic reticulum (SER). In APs RER can be found in both apical and basal processes (Figure 2). ER is an interconnected tubular network of membranes that are in continuity with the nuclear envelope. This feature is relevant in the context of APs, as they move the nucleus in concert with cell cycle during INM (Taverna et al., 2016; Taverna and Huttner, 2019). It would therefore be interesting to know if any secretory or signaling functions of the ER show cell cycle-dependency as a consequence of nuclear movement. The RER is the starting point of the secretory pathway, where nascent proteins are transported while being translated by ribosomes (hence the name of rough ER).

Proteins undergo rounds of preliminary modifications and, if not correctly folded or modified, they are delivered back to the cytoplasm for proteasome degradation (Haynes et al., 2004; Ninagawa et al., 2021). Failure to degrade misfolded proteins causes ER stress which is in turn associated with apoptosis. There are several ways through which RER is actively involved in proteostasis; one is the unfolded protein response (UPR) that in mammals is mediated by three pathways that put the ER in communication with the nucleus: IRE1a, PERK and ATF6 are transmembrane receptors on the RER surface that sense misfolded proteins and after activation upon BiP/GRP78 dissociation, they trigger the UPR response through different pathways (Silvestre et al., 2009; Chao et al., 2014; Passemard et al., 2019).

#### ER as sensor for stress

ER proteostasis revealed to be crucial during cortical development. Laguesse and colleagues showed that an upregulation of the UPR through the activation of the PERK-eIF2a-Atf4 signaling, caused by KO of Elp3 (a member of the elongator complex) leads to an increase in direct production of neurons from APs, at the expenses of the indirect neurogenesis pathway (Laguesse et al., 2015). The depletion of IPs leads to a microcephalic phenotype in mice bearing the mutation (Laguesse et al., 2015). These observations elegantly highlight the relevance of ER stress response and proteostasis for cell fate specification and lineage progression in the developing brain.

Other ER stressors that come from the external environment, such as viruses can interfere with ER proteostasis and lead to aberrations in the development of the cortex. This is the case of Zika virus for example, that not only induces massive cell death but also causes ER stress, triggering UPR and contributing ultimately to primary microcephaly development in newborns (Wang and Ling, 2016). Another trigger for UPR is alcohol consumption by pregnant women, that directly exert an effect on the newborn developing brain, through epigenetic alterations, perturbation of calcium homeostasis and generation of abnormal protein triggering UPR (Ji, 2012). Collectively, the involvement of the ER-related UPR response in microcephaly pathogenesis leads to interesting questions as to which is the role of UPR in cell fate switch in physiological conditions (Tomás et al., 2012; Casey et al., 2016; Passemard et al., 2019). One could speculate that during fate transition, in parallel to the radical changes in the transcriptional landscape, cells need to completely renew also their transcriptome and proteome *repertoire*. In that context, UPR might serve the purpose of implementing in an efficient and coordinated way those changes.

In NPCs, the ER has also been reported to form a diffusion barrier with a role in the asymmetric inheritance of mono- and polyubiquitylated proteins (Bin Imtiaz et al. 2022): though the relevance in terms of effect on progenitor cells identity following disruption has not been fully elucidated yet, future research on the topic will help clarifying whether the ER could affect NPCs identity by regulating the partitioning and segregation between old or newly synthetized proteins.

### ER-Golgi intermediate compartment

The ER-Golgi intermediate compartment (ERGIC) is a vesiculotubular organelle that mediates the anterograde and retrograde transport between ER and GA. In polarized cells, such as neurons, the ERGIC compartment has been proposed to allow the GA derived-compounds to communicate with the most distant dendritic compartments(Breuza et al., 2004; Appenzeller-Herzog and Hauri, 2006; Zhang et al., 2009; de los Ángeles Juricic Urzúa et al. 2021). Though in neurons it has been recently reported to be composed of both stationary and mobile compartments, the extent of ERGIC polarization in NPCs is currently not known.

Given the extreme elongation of APs (and bRG), one could speculate that the ERGIC compartment might have a role in helping the ER to sustain the complex process of protein maturation and modification that allows membranes and associated proteins to be delivered to distal locations, such as the basal process and/or the basal end foot.

### The Golgi apparatus

The GA is the first identified traffic organelle whose main function is adding glycan modifications to secretory pathway components. The GA is composed of multiple cisternae packed together to form a stack. Cisternae progress from the *cis*-Golgi, closer to the ER, through the *medial*- and then the *trans*-Golgi, where modified proteins or lipids enter the trans Golgi network (TGN) to be directed to their destination. Proteins arriving from the ER and ERGIC, are already partially modified by the ER with the addition of a core oligosaccharide chain. In the *medial*-Golgi a series of glycosyltransferase add sugars and by the time the protein reaches the exit site, the TGN, it is functional and ready to be delivered to the plasma membrane. In mammalian cells GA stacks are connected to form the Golgi ribbon (D’Souza et al., 2021). Interestingly, in APs the GA is not forming the typical Golgi ribbon but it exist as separated stacks (Figure 1) (Taverna et al., 2016), very much resembling the GA organization in *Drosophila* (Gosavi and Gleeson, 2017; Fujii et al., 2020).

The communication and transport of cargoes between the GA stacks and other trafficking organelles is dependent on the actin and microtubule cytoskeleton that interact with GA-resident structural proteins (Silvestre et al., 2009; Bowen et al., 2017; Passemard et al., 2019). GA interaction with the cytoskeletal elements is necessary not only for its integrity, but also for GA disassembly, a process that normally happens during the G2 phase of the cell cycle, when GA disassembly must occur to allow the cell to enter mitosis (Ayala and Colanzi, 2017). Blocking GA fragmentation results in G2 arrest, as a consequence of the so-called Golgi mitotic checkpoint (Sütterlin and Colanzi, 2010). Using depolymerizing drugs it is possible to actively induce fragmentation of GA at any time point of the cell cycle making the complex not functional anymore (Dinter and Berger, 1998; Breuza et al., 2004). In both situations, physiological or pharmacologically forced, most proteins and enzymes that were resident of the Golgi stacks, move to the ER and ERGIC compartments (Kemal et al., 2022).

#### Intracellular architecture of the Golgi apparatus: from neurons to NPCs

GA shows a polarized distribution in polarized cells, as beautifully shown in neurons, where it is found at the somatodendritic compartment while it is typically absent from the axon (Britt et al., 2016; Koppers and Farías, 2021). In pyramidal neurons the GA extends in the apical dendrite; this extension is crucial for cell polarization, axon specification and dendrite growth and is dependent on Ube3a, a gene found to be mutated in Angelman syndrome (AS) (Condon et al., 2013), a neurodevelopmental disease associated with severe learning disabilities. KD of Ube3a in mouse model of AS has been reported to be linked to defects in synaptic development and plasticity. Ube3a is a ubiquitin ligase that can target GA specific proteins. Interestingly, it has been hypothesized that in cortical neurons of AS mouse model, the link between Ube3a and GA resides in the under-acidification of GA cisternae leading to reduced protein sialyation (Condon et al., 2013).

Dendrites also contain Golgi outposts (GO), that ensure local and activity dependent modifications of proteins and lipids far away from the cell body (Bowen et al., 2017).

In APs the Golgi complex displays a non-canonical organization as (i) it shows a strong sub-compartmentalization, as it is only present in the apical process and it is dynamically reorganized during interkinetic nuclear migration (INM) (ii) it is not pericentrosomal (iii) it shows stacks but lacks the ribbon-like structure typically found in mammalian cells (iv) the stacks are organized perpendicular to the apicobasal axis, with the TGN surface parallel to the basolateral PM. Interestingly, GA is reorganized upon AP to BP fate transition, and in BPs GA is both pericentrosomal and perinuclear and shows a typical ribbon-like structure (Taverna et al., 2016; Taverna and Huttner, 2019) (Figure 2). These characteristics render the GA organization and re-organization quite unique and leave open several questions regarding the structure/function relation of such a peculiar architecture. We here discuss salient points and mention relevant questions that are still open.

#### Mechanisms and dynamics of GA confinement in the apical process

The confinement of the GA to the apical process is of great interest in light of INM. INM is a process of nuclear movement that is cell cycle dependent: immediately after mitosis at the apical surface, an AP moves the nucleus from the apical to the basal side of the VZ during G1, undergoes S-phase at the basal most part of the VZ and then in G2 moves the nucleus back to the apical surface for a new round of mitosis. During all phases of the cell cycle the GA is apical to the nucleus, with a small portion localized close to it (Taverna et al., 2016; Xie et al., 2018; Brault et al., 2022). This dynamic organization poses two questions: which are the mechanisms that allow the apical confinement of the GA and which are the structural and functional effects of nucleokinesis on the GA. The apical confinement of the GA is maintained via the activity of PITPNA and PITPNB, two phosphatidylinositol 4-phosphate transfer proteins involved in the synthesis of PI4P on the Golgi membrane. The PI4P pool recruits GOLPH3 which serves as an adaptor to link the cisternae to the actin cytoskeleton (Xie et al., 2018). As for the effect of nucleokinesis, live imaging experiments have shown that the GA in APs is stretched and elongated in G1, reaches it maximum apical-to-basal extension in S, and then it is compressed in G2, in concert with the basal-to-apical nuclear migration (Xie et al., 2018; Passemard et al., 2019). This accordion-like dynamics appears to be a peculiar feature of AP and they are intriguing in light of data linking mechanical forces applied on the GA to traffic from the GA (Guet et al., 2014). Indeed, since applying forces decreases vesicle budding from the GA, it would be interesting to understand if cycles of stretching and compression of the GA in AP in concert with the cell cycle result in a cell cycle dependent regulation of GA trafficking.

#### GA distribution and plasma membrane architecture

The confinement of the GA to the apical process has consequences for the organization and sub-compartmentalization of the basolateral plasma membrane of APs. By using single cell labeling combined with ConA and WGA (two lectins that specifically recognize ER- and GA-derived glycans) it was found that the basolateral plasma membrane of APs is subdivided in two domains that differ in their glycosylation state (Taverna et al., 2016). The apical process traversing the VZ contains both ER and GA-derived glycans while the basal process traversing SVZ and CP contains ER-derived glycans only. Interestingly, the glycans polarization appears to mirror the organelle polarization and asymmetry along the apico-basal axis of the cell (Taverna et al., 2016) (Figure 2). The interesting question is if and how the differential localization of the organelles and PM components could influence cellular identity. An intriguing possibility to explore is that the type of glycosylation impacts the activation and functionality of receptors, their delivery to the plasma membrane (or to specific sub compartments) and/or their signaling pathway, thus affecting cellular behavior and fate choice.

#### Intracellular architecture of fate transition

**Figure 3:**
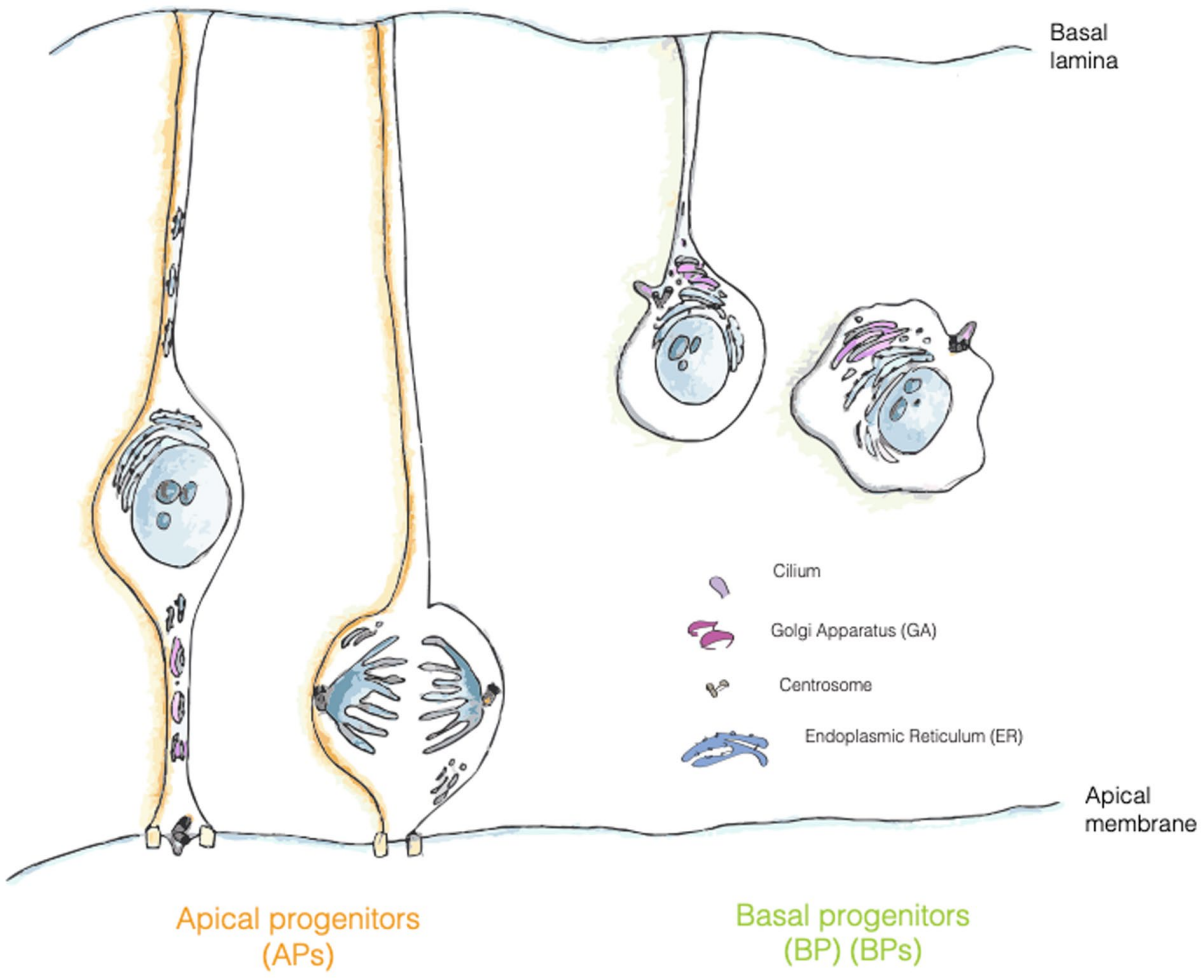
Localization of trafficking organelles in APs and BPs. Golgi apparatus (GA, pink) is formed by separate stacks localized in the apical process of APs and fragmented in mitotic AP, whereas in bRG and IPs it has a more canonical position and organization in the cell. Centrosome is represented in grey, RER in turquois and ciliary membrane is painted in purple.

AP-to-BP fate transition closely resembles an epithelial to mesenchymal transition (EMT). As in EMT, the generation of a BP entails a deep reorganization of the intracellular compartments, in particular of the cilium/centrosome and of the GA. In newborn BPs before delamination, the cilium is re-positioned from an apical to a basolateral location (Wilsch-Bräuninger et al., 2012; Taverna and Huttner, 2019) (Figure 3). Interestingly, delamination is also paralleled by a reorganization of the GA, that became pericentrosomal (Taverna et al., 2016; Taverna and Huttner, 2019) (Figures 1, 3). It is not yet fully clear if this reorganization is a consequence of the fate transition, or if it can also be a cause driving fate transition. On the relation between traffic and fate specification, Brault and colleagues have demonstrated that post-Golgi traffic mediated by Rab6 is crucial for delamination and BP generation. The data show that interfering with Rab6 traffic increases the number of BPs, thus suggesting that traffic might directly influence fate (Brault et al., 2022).

#### GA and centrosome: a long-lasting friendship story

In mammalian cells the GA is physically and functionally associated with the centrosome (Sütterlin and Colanzi, 2010). Despite the tight relationship, the pericentriolar positioning of the GA doesn’t appear to be fundamental for the secretory function of the organelle, in fact, organisms such as *Saccharomyces cerevisiae* or *Drosophila*, whose GA is not close to the centrosome still retain the ability to process and transfer proteins through the secretory pathway (Sütterlin and Colanzi, 2010). What is then the functional relevance for such an association? This association appears to be crucial for ciliogenesis, cell polarization and cell migration. As for ciliogenesis, the loss of GA proteins IFT20 and Rab8 impairs cilia formation. Of note, in the context of neurogenesis it has been demonstrated that in a portion of progenitors the ciliary membrane is asymmetrically inherited from the mother cell (Paridaen et al., 2013). This observation implies that at least half of newborn NPCs should rely on *de novo* ciliogenesis, that might depend on GA traffic routes, as suggested for work on cells in culture (Wilsch-Bräuninger et al., 2012; Witzgall, 2018; Diaz et al., 2020).

In general, APs offer an interesting paradigm regarding the centrosome/GA reciprocal regulation, as in interphase the GA and the centrosome are not physically associated (Taverna et al., 2016). The question arises as to whether they are still functionally associated, or if APs are lacking part of the regulation impinging on the GA/centrosome axis (Sütterlin and Colanzi, 2010).

### Function(s) of the Golgi apparatus

#### Golgi as secondary microtubule organizing center

The GA can nucleate microtubules, thus serving as non-centrosomal microtubule organizing centre (MTOC) (Yanagida et al., 2012; Gavilan et al., 2018). The GA-MTOC was shown to be relevant for directional protein transport (Zhu and Kaverina, 2013). For example, in *Drosophila* sensory neurons GA and Golgi outposts, have been observed to be necessary for dendrite branching. Microtubules nucleating from GA are emanated in an anterograde direction providing a directionality to transport and trafficking (Ori-McKenney et al., 2012). MTOCs are of crucial importance in NPCs as they regulate and fine tune the architecture and function of the cytoskeleton, affecting spindle dynamics and orientation, and in turn fate choice. In APs the distance between the Golgi stacks and the basolateral PM is only few microns, it would therefore be interesting to understand if the GA in APs serves as MTOC, and is so, how the GA-MTOC affects the directionality and efficiency of traffic.

#### Golgi as a stress sensor

In the last fifteen years GA emerged for its own role in proteostasis; it was already known the ability of the ER to respond to stressors and act as a cell sensor, triggering a response to restore homeostasis. It is now becoming clear that also the GA, in addition to its several roles, can act as a sensor to changes in pH that may affect its integrity and secretory functions, as well as to increased or decreased fraction of mature glycans. GA fragmentation-inducing drugs, such as brefeldin A, golgicide A or monensin, trigger a stress response mediated by ARF4 and CREB3. CREB3 is a leucine zipper transcription factor that, upon activation through proteolytic cleavage, translocates from the Golgi membranes to the nucleus, where it activates its target pro-apoptotic genes. CREB3 pathway is only one of the three Golgi-associated stress pathways known, the other two being TFE3 or HSP47 mediated. During stress responses TFE3 translocates to the nucleus, where it regulates, among others genes, glycosylation genes involved in sialylation and fucosylation (Sasaki et al., 2019; D’Souza et al., 2021). HSP47 pathway, identified by Miyata et al. (2013) and Sasaki et al. (2019), is activated by a different inducer of GA stress (BenzylGalNAc) which causes reduction of mucin-type glycosylation.

Recently, a fourth route that is triggered by increased or decreased proteoglycans capacity of the cell has been explored. Upon stress induced by the glycans themselves, GA starts a response ultimately leading to the activation of glycosylation enzymes for proteoglycans (Sasaki et al., 2019). GA has also been found to respond to DNA HR repair (Galea et al., 2022): in HeLa cells RAD51C, a regulatory HR protein, shuttles from the Golgi membranes to the perinuclear region following a DNA stress. RAD51 is linked to the golgin GM130, a multifunctional protein involved in GA structural maintenance. GM130 expression is under the control of TFE3 and was reported to be defective in some neuromuscular syndromes present in concert with microcephaly (Dinter and Berger, 1998; Passemard et al., 2019).

#### GA as a central hub for glycosylation

Proteins and lipids traveling through the GA are modified via the addition of sugars, reflecting one of the main functions of the GA: glycosylation. Glycosylation is a post translational modification that is crucial for regulating proteins and lipids function for signaling and/or structural purposes. Integrins are an excellent example of N-glycans in neurodevelopment; integrins are receptors found on the cell surface that require the presence of N-linked oligosaccharides to form functional dimers. These proteins are involved in a plethora of functions, such as cell migration and polarization in NPCs (Taverna and Huttner, 2003). Pathological changes in glycosylation are present in several diseases, ranging from neurodevelopmental and neurological disorders, to autoimmune diseases and cancer (Chang et al., 2018; Reily et al., 2019; Linders et al., 2020; D’Souza et al., 2021).

### Neurodevelopmental diseases associated with trafficking pathway defects

Golgipathies is a term used to define all the diseases associated with defects in the trafficking pathway, in particular of the Golgi apparatus. Over 40% of GA-related genes known to be associated with diseases show central and peripheral nervous system clinical manifestations (Jaeken and Matthijs, 2001; Freeze et al., 2014, 2015) (see Table 1). This might be due to the extensive use of the trafficking pathway in neurons and glia, where traffic is used for synaptic transmission, for receiving signals or even to form the myelin sheet. Furthermore, evidences suggest that aberrations in ER- and GA-dependent trafficking could contribute to the pathophysiology and progression of several neurodegenerative diseases, however, in this case it is not yet clear if the aberrations are a cause or a consequence of disease (Caracci et al., 2019).

**Table 1:** Trafficking-related structural proteins and enzymes found mutated in neurodevelopmental disorders.

Protein	Clinical manifestations	References
Structural proteins		
TRAPPC9	Autosomal recessive mental retardation MRT13	Mir et al. (2009)
TRAPPC12	Progressive childhood encephalopaty	Milev et al. (2017)
TRAPPC6B	Neurodevelopmental disorder with microcephaly, epilepsy and autistic features	Marin-Valencia et al. (2018)
CDC42	Takenouchi-Kosaki syndrome	Flynn et al. (2021)
LGALS3BP	Defects in neurodevelopment in de novo mutations	Kyrousi et al. (2021)
GM130	Neuromuscular syndrome with microcephaly	Shamseldin et al. (2016)
RABs	Several cancer types, Charcot Marie Tooth syndrome, Warburg micro syndrome	Banworth and Li (2018)
VPS13B	Cohen syndrome	Seifert et al. (2011)
ARF1	Brain abnormality	Ge et al. (2016)
ARF(3)	*De novo* mutation with developmental delay, epilepsy and brain abnormalities	Sakamoto et al. (2021)
ARFGEF	Autosomal recessive periventricular heterotropia with microcephaly	Xu et al. (2022)
COG complex	CDGII, mild to severe neurological impairment, microcephaly, mental retardation, cerebellar atrophy, hypotonia	Climer et al. (2015)
Enzymes		
DPM2	CDG I, muscular dystrophy-dystroglycanopathy syndrome	Barone et al. (2012)
ALG1	CDG I, with broad clinical spectrum of neurodevelopmental disease	Ng et al. (2016)
ALG3	CDG I, severe developmental delay, epilepsy, cortical atrophy cerebellar vermis hypoplasia and ocular impairment	Farolfi et al. (2021)
ALG11	CDG I, neurodevelopmental defects, psycomotor disabilities and epilepsy	Haanpää et al. (2019)
SLC35A	CDG II, with severe ID and POM	Ng et al. (2013)
POMT1, POMT2	O-mannose disorders, Walker-Warburg syndrome	Vajsar and Schachter (2006)

#### Disease genes associated with GA structure, integrity and functional maintenance

The structural integrity of the GA and its organization in stacks are maintained thanks to proteins such as golgin-45, GM130 and Giantin, GRASPs and the small GTPases RABs, ARFs, ARLs and TRAPPs (see Table 1). The TRAPP complex activates RAB1 which in turn recruits golgins and GM130 at the cis-Golgi level, to ensure vesicle tethering; furthermore, TRAPPC9 is also involved in the association between COPII-coated vesicles and dynein for the movement of vesicles on the cytoskeleton. Loss of function mutations of TRAPPC9 have been identified in Autosomal recessive mental retardation 13 (MRT13), that shows as a clinical manifestation moderate to severe post-natal onset microcephaly (POM), thinning of the corpus callosum, a peculiar facial appearance, hypotonia and obesity (Mochida et al., 2009). TRAPPC12 mutations are associated with progressive childhood encephalopathy, progressive microcephaly and developmental defects. Another example is represented by Takenouchi-Kosaki syndrome, caused by mutations in the GTPase CDC42 which regulates bidirectional Golgi transport, cargo sorting and COPI formation; affected patients show a wide range of phenotypes, including ID, POM or congenital microcephaly (Rasika et al., 2018). Recent work showed the relevance of LGALS3BP in the generation and positioning of apical and basal NPCs (Kyrousi et al., 2021). LGALS3BP a cancer biomarker, is a galectin 3 binding protein, a secreted protein that interacts in the extracellular matrix with integrins, fibronectins and other components. Such example provides further evidence that the trafficking pathway, of which the GA is one of the central players, can influence brain development in multiple ways, affecting cellular components such as receptors, as well as the extracellular *milieu* (i.e., ECM components and interacting factors, secreted signals, etc.). Of note, receptors and ECM components are typically highly glycosylated proteins, suggesting the possibility that the change in the glycosylation state of these components might be one of the pathophysiological mechanisms underlying neurological manifestations.

#### Disease genes associated with the GA glycosylation machinery

Though several diseases have been found to be associated with GA secretory and trafficking properties, we’d like to focus on one specific group of rare diseases called congenital disorders of glycosylation (CDGs). CDGs are a group of rare diseases associated with defects in glycosylation and aberrations on the ER and GA-trafficking pathways leading to (Jaeken and Casaer, 1997; Matthijs et al., 1997; Jaeken and Matthijs, 2001; Grunewald, 2002; Freeze and Ng, 2011; Freeze et al., 2015; Chang et al., 2018; Linders et al., 2020; Paprocka et al., 2021). CDGs are typically due to either mutations in ER and GA structural components or in ER and GA glycosylation enzymes. In the latter case traffic is indirectly affected most likely because of the activation of glycosylation quality check and/or because of defective protein folding (see also the section on UPR).

N-glycosylation associated CDGs, that are easier to identify, can be subdivided in Type I, caused by defects in the association of the first oligosaccharides to the lipid anchor at the level of the ER or in the cytoplasm; Type II CDGs are instead associated with defects at the level of the ER or Golgi in the glycosylation process or on the remodeling of N-glycans. Though CDGs affect usually different organs of the body, the most severe effects are quite often noticed in the central nervous system at birth, probably because of the relevance of trafficking in neurodevelopment. This observation might not come to a surprise, as both neurons and progenitors are highly polarized cells, relying on heavy supply of membrane components, a feature that can make them particularly sensitive to any perturbation of the secretory pathway.

Severe congenital malformations associated with CDGs include microcephaly, macrocephaly, ventriculomegaly/hydrocephalus, myelination disorders, corpus callosum anomalies and brain atrophy (Paprocka et al., 2021) (see Table 1). On the contrary O-linked CDGs, conditions that affect secreted protein, are often associated with neuronal migration defects, as illustrated by muscular dystrophy-dystroglycanopathy (MDDG) syndrome (Nguyen et al., 2013). This could suggest that neuronal migration is dependent on accurate extracellular matrix deposition; on the other hand, plasma membrane-bound glycans seem to affect intrinsic transcriptional programs, thereby influencing cell identity probably because most membrane glycans are receptors involved in signaling pathways.

Indeed, defects on structural proteins of the ER or of the GA impair the whole trafficking pathway, resulting in aberrations in how and where enzymes are partitioned. Yet another way to perturb the glycosylation process is through alteration of the sugar supply or directly of the enzymes involved in glycosylation. Biosynthesis of N-glycans starts in the ER, where the family of ALG enzymes sequentially add sugar moieties to dolichol phosphate (Dol-P). Dol-P is synthesized from GDP-Mannose by the multi subunit complex DPM (Dol-P-Man) (Lauc and Trbojević-Akmačić, 2021). Consistently, microcephaly and neurodevelopmental defects are also present in CDG patients with mutations on DPM2, ALG11, SLC35A, ALG1 and ALG3. SLC35A2 belongs to the SLC35A family of sugar transporters and mutations in the X-linked UDP-galactose transporter gene leads to CDGs as they cause defective galactosylation of glycoconjugates in GA (Quelhas et al., 2021). ALG1, 3 and 11 enzymes are a b1,4 mannosyltransferase, a1,3 mannosyltransferase and an asparagine linked glycosylation protein 11 respectively, and as mentioned they are involved in the glycosylation process at the ER level (Rasika et al., 2018; Paprocka et al., 2021).

Along the glycosylation pathway, N-glycans are first transferred to the Asn residue of a protein by OST (oligosaccharyl-transferase) and then enter the GA, where further remodeling of the sugar chain is operated by mannosidase and/or fucosyltransferases (i.e., FUT). For example, mutations in the MGAT2 gene, that encodes for an acetylglucosaminyltransferase, is associated in CDG type II with neurological defects and hydrocephalus (Rasika et al., 2018). Cerebellar defects are instead observed related to mutations to another GA enzyme, INPP5E (Rasika et al., 2018). Ng et al. (2018) described three unrelated children with FUT8 mutations associated with growth and developmental retardation, neurological impairments and respiratory complications among the clinical manifestations.

At the level of GA complex-type glycans are formed. N-glycans can undergo further modification via addition of fucose, sialic acid, sulfate residues that increase the glycans’ complexity (Lauc and Trbojević-Akmačić, 2021). Thus, disrupting GA at the structural or functional level can lead to several different outcomes that translate in different clinical manifestations of CDG.

Alternatively, the O-glycosylation pathway can happen in the GA, through a different set of glycosyltransferases (i.e., POMT1,2) (Vajsar and Schachter, 2006) that allows the addition of sugars to the serine or threonine of molecules that are mainly destined to the extracellular environment. POMT1 and POMT2 mutations have been observed in patients with muscular dystrophy and intellectual disability. The number of congenital disorders with neurological manifestations classified as CDGs is steadily increasing in the last years, clearly highlighting the relevant connections between the homeostasis of trafficking pathway and neurodevelopment. Of note, a precise mechanistic explanation of how traffic and associated glycosylation affect neural stem cell behavior and in turn brain development is still lacking.

We think this class of diseases will provide an excellent opportunity for researchers to dig further into the deep secrets of the trafficking and Golgi dynamics in neurons and in neural progenitors where the journey of the nervous system development starts.

### Concluding remarks

In this review we summarize what is known up to date about the role of trafficking and in particular of Golgi apparatus in neurodevelopment. First of all, we summarized the basics of neurodevelopment, introducing the relevant cell types that concur in defining the neocortex in mammals. Lot of work has been done in unraveling the pathways underlying trafficking in neurons, but lot has still to be done in the context of neural progenitors and neurodevelopment. There is no doubt that research done on neurons provide a helpful access route to studies on NPCs, in particular on apical progenitors, as they (very much like neurons) are a case of extreme subcellular and architectural polarization and asymmetry. A question still left unanswered is the (possible) relation between cell identity and the Golgi complex, along with GA-dependent glycosylation, in light of the peculiarities of GA organization, localization and dynamics in APs. The occurrence of diseases, such as CDGs, where alterations in glycosylation are paralleled by strong neurodevelopmental manifestations, provide a real-life example of the functional link between the trafficking and glycosylation machinery and neurodevelopment. By leveraging CDG’s genetics researchers might hope to unravel the basic principles linking sugar modifications with NPC behavior and brain development in health and disease. The existence of a second route for protein and lipid glycosylation, could direct future research to unravel yet unknown functions of trafficking organelles in different processes, including regulation of cell polarity cues in the context of neurodevelopment.

In a broader perspective, given that the heterogeneity and specificity of the sugar components of the added oligosaccharide chain make glycosylation highly combinatorial, it would be interesting to understand if a sugar-code exists, and if so, how it is exploited during brain development and evolution to affect and influence fate choice and cell identity. In this context is worth remembering that also lipids can be glycosylated, further increasing the level of complexity potentially reached by the sugar-code. A relevant step in this direction has been recently made by Lee et al. (2020) by showing that the glycome signature diverge spatially and temporally in mouse and human brain samples.

The advent of new techniques for lineage tracing and glycan analysis, along with the possibility to tightly control cell polarity and microenvironment using microfabrication, will certainly increase access and interest to this research field. At the same time, when considering pathological conditions, the possibility to use patient derived iPSC to grow brain organoids and organs on chip, will help recapitulating *in vitro* what happens *in vivo*, opening new avenues in our understanding of physiological processes and opening up, in future, to therapeutical interventions.

## Author contributions

All authors listed have made a substantial, direct, and intellectual contribution to the work and approved it for publication.
