# Supplementary material for: Intracellular traffic and polarity in brain development
Source: Front Neurosci. 2023 Oct 4;17:1172016. doi: 10.3389/fnins.2023.1172016 (PMC10583573; doi:10.3389/fnins.2023.1172016)
Supplement: Supplementary file 1 [file Data_Sheet_1.PDF]

	Protein	Clinical manifestations	References
Structural proteins	TRAPPC9	Autosomal recessive mental retardation MRT13	(Mir et al. 2009)
	TRAPPC12	Progressive Childhood encephalopaty	(Milev et al. 2017)
	TRAPPC6B	Neurodevelopmental disorder with microcephaly, epilepsy and autistic features	(Marin-Valencia et al. 2018)
	CDC42	Takenouchi-Kosaki syndrome	(Flynn et al. 2021)
	LGALS3BP	Defects in neurodevelopment in de novo mutations	(Kyrousi et al. 2021)
	GM130	Neuromuscular syndrome with microcephaly	(Shamseldin et al. 2016)
	RABs	Several cancer types, Charcot Marie Tooth syndrome, Warburg micro syndrome	(Banworth and Li 2018)
	VPS13B	Cohen syndrome	(Seifert et al. 2011)
	ARF1	Brain abnormality	(Ge et al. 2016)
	ARF(3)	de novo mutation with developmental delay, epilepsy and brain abnormalities	(Sakamoto et al. 2021)
	ARFGEF	Autosomal recessive periventricular heterotropia with microcephaly	(Xu et al. 2022)
	COG complex	CDGII, mild to severe neurological impairment, microcephaly, mental retardation, cerebellar atrophy, hypotonia	(Climer, Dobretsov, and Lupashin 2015)
Enzymes	DPM2	CDG I, muscular dystrophy- dystroglycanopathy syndrome	(Barone et al. 2012)
	ALG1	CDG I, with broad clinical spectrum of neurodevelopmental disease	(Ng et al. 2016)
	ALG3	CDG I, severe developmental delay, epilepsy, cortical atrophy cerebellar vermis hypoplasia and ocular impairment	(Farolfi et al. 2021)
	ALG11	CDG I, neurodevelopmental defects, psycomotor disabilities and epilepsy	(Haanpää et al. 2019)
	SLC35A	CDG II, with severe ID and POM	(Ng et al. 2013)
	POMT1,POMT2	O-mannose disorders, Walker-Warburg syndrome	(Vajsar and Schachter 2006)
