# Supplementary material for: Intracellular traffic and polarity in brain development
Source: Front Neurosci. 2023 Oct 4;17:1172016. doi: 10.3389/fnins.2023.1172016 (PMC10583573; doi:10.3389/fnins.2023.1172016)
Supplement: Supplementary file 2 [file Data_Sheet_2.PDF]

## Contribution to the field

Neurons forming the human brain are generated during embryonic development by neural stem and progenitor cells (NPCs) via a process called neurogenesis. A crucial feature contributing to neural stem cell morphological and functional heterogeneity is cell polarity. Cell polarity is built and maintained thanks to the interplay between polarity proteins and polarity-generating organelles. In the last decades our knowledge on the role of polarity in neural stem and progenitor cells have increased tremendously. Similar progress has been made in the study of traffic in neurons. However, the role of traffic and associated glycosylation in NPCs is relatively underexplored, and researchers started to venture in that direction only recently.

With our review, we highlight recent advances in this field and discuss future directions of research, including the possibility of leveraging the genetics of congenital disorders of glycosylation to gain insight into the role of traffic and glycosylation in brain development in health and disease.
